# Hepatic steatosis induced by nicotine plus Coca-Cola™ is prevented by nicotinamide riboside (NR)

**DOI:** 10.3389/fendo.2024.1282231

**Published:** 2024-05-02

**Authors:** Juan Carlos Rivera, Jorge Espinoza-Derout, Kamrul M. Hasan, Jocelyn Molina-Mancio, Jason Martínez, Candice J. Lao, Martin L. Lee, Desean L. Lee, Julian Wilson, Amiya P. Sinha-Hikim, Theodore C. Friedman

**Affiliations:** ^1^ Division of Endocrinology, Metabolism and Molecular Medicine, Department of Internal Medicine, Charles R. Drew University of Medicine and Science, Los Angeles, CA, United States; ^2^ David Geffen School of Medicine at University of California, Los Angeles, Los Angeles, CA, United States; ^3^ Biostatistics Department, UCLA Fielding School of Public Health, Los Angeles, CA, United States

**Keywords:** nicotine, high-fructose corn syrup, hepatic steatosis, nicotinamide riboside, NAD^+^

## Abstract

**Introduction:**

Cigarettes containing nicotine (Nic) are a risk factor for the development of cardiovascular and metabolic diseases. We reported that Nic delivered via injections or e-cigarette vapor led to hepatic steatosis in mice fed with a high-fat diet. High-fructose corn syrup (HFCS) is the main sweetener in sugar-sweetened beverages (SSBs) in the US. Increased consumption of SSBs with HFCS is associated with increased risks of non-alcoholic fatty liver disease (NAFLD). Nicotinamide riboside (NR) increases mitochondrial nicotinamide adenine dinucleotide (NAD^+^) and protects mice against hepatic steatosis. This study evaluated if Nic plus Coca-Cola^™^ (Coke) with HFCS can cause hepatic steatosis and that can be protected by NR.

**Methods:**

C57BL/6J mice received twice daily intraperitoneal (IP) injections of Nic or saline and were given Coke (HFCS), or Coke with sugar, and NR supplementation for 10 weeks.

**Results:**

Our results show that Nic+Coke caused increased caloric intake and induced hepatic steatosis, and the addition of NR prevented these changes. Western blot analysis showed lipogenesis markers were activated (increased cleavage of the sterol regulatory element-binding protein 1 [SREBP1c] and reduction of phospho-Acetyl-CoA Carboxylase [p-ACC]) in the Nic+Coke compared to the Sal+Water group. The hepatic detrimental effects of Nic+Coke were mediated by decreased NAD^+^ signaling, increased oxidative stress, and mitochondrial damage. NR reduced oxidative stress and prevented mitochondrial damage by restoring protein levels of Sirtuin1 (Sirt1) and peroxisome proliferator-activated receptor coactivator 1-alpha (PGC1) signaling.

**Conclusion:**

We conclude that Nic+Coke has an additive effect on producing hepatic steatosis, and NR is protective. This study suggests concern for the development of NAFLD in subjects who consume nicotine and drink SSBs with HFCS.

## 1 Introduction

Cigarette smoking continues to be one of the most important causes of morbidity and mortality in the United States (1). In 2020, it was reported that 19% of United States adults consume any tobacco product (2). Moreover, new ways to deliver nicotine (Nic) one of the principal components of cigarettes are increasing, such as electronic cigarettes, cigars, and little cigars, which are popular among the young population (3). Thus, smoking/vaping continues to be a public health issue even though several restrictions have been implemented to reduce nicotine consumption. Smoking is a major risk factor for cardiovascular and/or metabolic disease including non-alcoholic fatty liver disease (NAFLD) (4). Nic has been shown to produce hepatic steatosis when administrated via intraperitoneal (IP) injection together with a second-hit such as a high-fat diet (HFD) (5, 6). Previously, we demonstrated that a key mechanism mediating hepatic steatosis consists of increased oxidative stress, liver lipogenesis, and decreased in the phosphorylation of the metabolic regulator AMP-activated protein kinase (AMPK) (5).

NAFLD is a multi-hit disease characterized by the accumulation of lipid droplets in liver cells (7) with a world-wide prevalence of NAFLD of 25% (8, 9). This is the consequence of the population’s current nonhealthy lifestyle, such as being sedentary and consuming a western diet (7). Among the dietary component of the western diet, sugar-sweetened beverages (SSBs) including soft drinks, have increased in consumption in the last years being a major source of added caloric sugar in both adults and youths (10–12). In the US, the principal sweetener of SSBs is high fructose corn syrup (HFCS), which is a compound of fructose and sucrose and is also present in processed food (13, 14). The major problem of HFCS in SSBs is the high caloric ingestion producing increased triglycerides and reduced HDL-C levels in the absence of the micronutrients (15). Additionally, HFCS and SSBs have been associated with insulin resistance, weight gain, metabolic syndrome, lipogenesis, hepatic diseases (16–19) and a higher risk of NAFLD and liver failure (20). At the molecular level, HFCS promotes lipogenesis through the activation of the master transcriptional factor, the sterol regulatory element-binding protein (SREBP1) by cleavage (SREBP1c) from full-size protein (21).

At present, there are limited treatments for NAFLD despite its high world-wide incidence. Nicotinamide adenine dinucleotide (NAD^+^) is a central molecule of the redox reactions and is involved in additional functions such as DNA repair, epigenetic modification, and aging (22). Cellular NAD^+^ concentrations are maintained by *de novo* synthesis from amino acid tryptophan, or the salvage pathways through the recycling of nicotinamide to produce NAD^+^. The salvage pathway can be supplemented by exogenous precursors (23, 24). Interestingly, NAD^+^ concentrations are depleted in mice fed with HFD, who develop NAFLD (25). In aged humans and mice, hepatic NAD^+^ levels are reduced that is a risk factor to develop NAFLD (26). Nicotinamide riboside (NR) is a precursor of NAD^+^ that reduces plasma triglycerides and total cholesterol levels, in turn preventing hepatic lipid accumulation in mice and humans (27–29). Sirtuin 1 (Sirt1), an NAD^+^-dependent enzyme, has a protective effect in NAFLD. Sirt1 promotes mitochondrial biogenesis through peroxisome proliferator-activated receptor coactivator 1-alpha (PGC1*α*) (30, 31). NR supplementation activates Sirt1 signaling (32), and activators of Sirt1 are promissory candidates for NAFLD treatment (33). The goal of the present study is to determine if the administration of Nic and HFCS in the oral consumption of Coca-Cola™ (Coke) with HFCS can cause hepatic steatosis and that can be protected by NR. In this study, we have elucidated some of the molecular insights of Coke with HFCS on hepatic steatosis.

## 2 Materials and methods

### 2.1 Mice

Adult male (10-week-old) C57BL/6J mice (22–24 g BW) purchased from Taconic Farms (Germantown, NY, USA) were housed (4–5 per cage) in a standard animal facility under controlled temperature (22 °C) and photoperiod (12-h light and 12-h dark cycle) with food and water *ad libitum*. Mice received twice-daily intraperitoneal (IP) injections of Nic (0.75 mg/kg body weight) which is equivalent to smoking two-pack a day or saline (vehicle) for 10 weeks as reported previously in our laboratory (5). Nic was maintained in a dark container to prevent light exposure. Additionally, mice in both groups received Coca-Cola^TM^ containing HFCS (Coke) (carbonate water, HFCS, caramel color, phosphoric acid, natural flavors, caffeine; 140 cal per 360 mL, 0 g fat, 45 mg sodium, 39 g carbohydrate, and 0 g protein) or sugar Coke (S-Coke) (carbonate water, cane sugar, caramel color, phosphoric acid, natural flavors, caffeine; 140 cal per 360 mL, 0 g fat, 45 mg sodium, 39 g carbohydrate, and 0 g protein), which is a special run of Coke containing sugar and absolutely no HFCS from a store in a neighborhood with a larger Jewish population prior to the holiday of Passover, or water in drinking bottles. To prevent hepatic damage and increase NAD^+^ levels, additional groups of mice were supplemented with twice daily IP injection of NR in saline solution (200 mg/Kg BW/day) or saline solution (vehicle) to the other groups with each injection having the same volume (200 μl). The details of the injections are summarized in **Supplementary Table 1**. Mice were individually weighed weekly and group food and liquid consumption were measured weekly. Mice fasted overnight before euthanization with a lethal injection of sodium pentobarbital (200 mg/kg BW) and the liver was removed. Pieces of the liver were either fixed in 2.5% glutaraldehyde (Sigma Aldrich, St. Louis, MO, USA) for high-resolution light microscopy and electron microscopy. For routine histological studies, we used 4% paraformaldehyde (PFA) (Fisher Scientific, Hampton, NH, USA) as described previously (5). Additional liver pieces were quickly removed snap-frozen in liquid nitrogen and stored at -80 °C for Western blot analysis. Animal handling and experimentation were in accordance with the recommendation of the current National Institutes of Health guidelines and were approved by the CDU and Lundquist Institute Animal Care and Use Committees (IACUC).

### 2.2 Histological analysis

Liver pathology was evaluated using conventional histological analysis on hematoxylin and eosin (H&E)-stained sections. Further evaluation of pathology was achieved by high-resolution light microscopy using glutaraldehyde-fixed, osmium tetroxide postfixed, epoxy-embedded, and toluidine blue-stained sections as described previously (5, 34). Quantitative analysis of hepatic steatosis was carried out in a blind fashion using a scoring analysis as described previously (35). The score considered parameters such as macrovesicular steatosis, microvesicular steatosis, hepatocyte hypertrophy, and inflammatory focus (**Supplementary Table 2**) (35). The histological features were analyzed by light microscopy Olympus Bx51 (Olympus, Tokyo, Japan) at 10x or 40x objectives in five different fields.

### 2.3 Transmission electron microscopy

For electron microscopy analysis, small pieces of glutaraldehyde-fixed livers were cut into small pieces, post-fixed in 1% osmium tetroxide, dehydrated in a graded series of ethanol, and embedded in Epon 812 as described previously (5, 34, 36). Embedded liver tissues were cut with an LKB ultramicrotome, stained with uranyl acetate and lead citrate, and examined with a Hitachi electron microscopy (Hitachi, Indianapolis, IN, USA).

### 2.4 Liver triglyceride quantification

Liver triglyceride (TG) content was quantified in the frozen samples. Liver tissues were homogenized with NP-40 buffer plus protease and phosphatase inhibitors (Sigma Aldrich, St. Louis, MO, USA). The supernatants were used to quantify the TG, using the commercially available Triglyceride Colorimetric Assay Kit (Cayman Chemical, Ann Arbor, MI, USA) following the manufacturer’s instructions.

### 2.5 Western blotting

Western blotting was performed using liver lysates as described previously (37, 38). Briefly, we separated proteins (60-100 *µ*g) in 10-12% SDS-PAGE in TRIS-Glycine-SDS buffer (Fisher Scientific, Hampton, NH, USA) to 100-120 V. Then, proteins were transferred to a Nitrocellulose membrane (Bio-Rad, Hercules, CA, USA) for 1 hour to 300 mAmp at 4°C in TRIS-Glycine buffer (Bio-Rad, Hercules, CA, USA). Next, membranes were stained with Ponceau S solution (Sigma Aldrich, St. Louis, MO, USA) for 5 minutes at room temperature, rinsed with distilled water, and blocked in 0.1% Tween-20, Tris Buffer Saline pH 7.4, and 5% non-Fat milk (blocking solution) for one hour at room temperature. We then incubated the membranes with the following first antibodies (diluted in blocking solution) including, rabbit polyclonal phospho-Acetyl-CoA Carboxylase (p-ACC) (1:1000) (3661; Cell Signaling Technology, Beverly, MA, USA), rabbit polyclonal phospho-AMP-activated protein kinase (p-AMPK) (1:1000) (2535; Cell Signaling Technology, Beverly, MA, USA), rabbit polyclonal HO-1 (1:2000) (AB13243; Abcam, San Francisco, CA, USA), mouse monoclonal Mitochondrial complex cocktail (1:3000) (AB110413; Abcam, San Francisco, CA, USA), rabbit polyclonal NAMPT (1:250) (AB45890; Abcam, San Francisco, CA, USA), rabbit polyclonal PGC1*α* (1:1000) (AB54481; Abcam, San Francisco, CA, USA), rabbit polyclonal Sirt1 (1:2000) (AB12193; Abcam, San Francisco, CA, USA), rabbit polyclonal SOD2 (1:500) (sc-30080; Santa Cruz Biotechnology, Santa Cruz, CA, USA), mouse monoclonal SREBP1 (1:500) (AB3259; Abcam, San Francisco, CA, USA), and rabbit polyclonal *β*-Actin (1:4000) (AB8227; Abcam, San Francisco, CA, USA). *β*-Actin was used as a loading control, all antibodies were incubated overnight at 4°C with constant shaking. On the next day, membranes were rinsed in TBS-Tween-20 and incubated with anti-mouse or anti-rabbit IgG secondary antibody (Abcam, San Francisco, CA, USA). Membranes were then rinsed TBS-Tween-20, and protein was recognized by chemiluminescence using ECL detection kits (Thermo Fischer Scientific, MA, USA) using the imaging system LI-COR Odyssey® XF (NE, USA). Band intensities were quantified using Image J software (NIH, USA).

### 2.6 Lipid peroxidation

We measured malondialdehyde (MDA) generation in liver tissue using the thiobarbituric acid substance assay (TBARS) following the manufacturer protocol (Cayman Chemical, MI, USA), as previously reported (39).

### 2.7 Statistical analyses

Statistical analyses were done using Prism 9.1.0 software (GraphPad Software, Inc). Data were shown as mean ± standard error of the mean (S.E.M.). Multiple comparisons were performed with One-way ANOVA and Two-way ANOVA, with Holm-Sidak’s as a post-test. To determine statistical differences between food and liquid consumption we compared the slope of the groups through an analysis of covariance (ANCOVA). Differences were considered significant if *p*<0.05.

## 3 Results

### 3.1 Bodyweight and caloric intake

**Figure 1:**
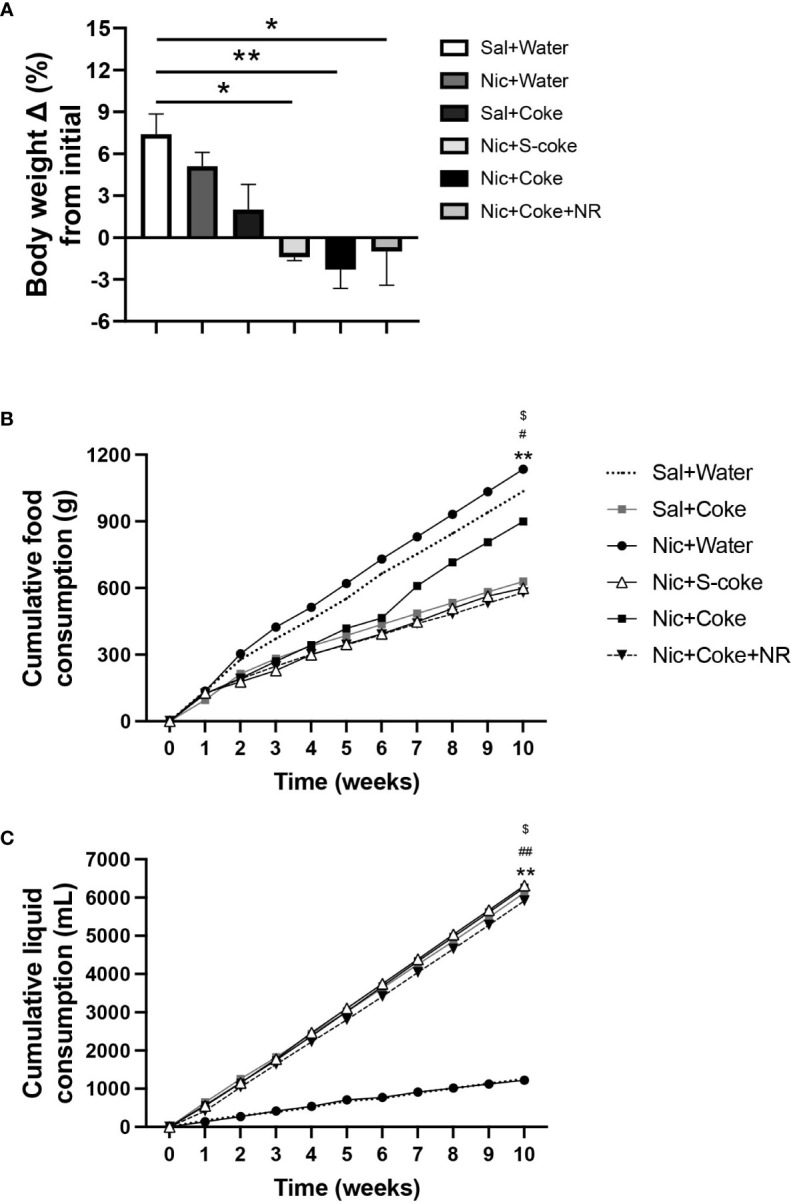
Changes in bodyweight and caloric intake in mice. Mice received twice-daily IP injections of Nic (0.75 mg/kg body weight) or saline for 10 weeks. Additionally, mice received Coca-Cola^TM^ containing sucrose (S-Coke) or HFCS (Coke), or water in drinking bottles. Mice received a twice NR IP injections (200 mg/Kg BW/day) and normal chow food. **(A)** Percentage change of the initial body weight difference at ten weeks of treatment. **(B)** Cumulative food consumption (g). **(C)** Cumulative liquid consumption (mL). Values are given as mean ± S.E.M., n= 4 - 5 per group. Statistical difference is indicated by *Sal+Water vs Nic+Coke (P< 0.05); ^$^Nic+S-Coke vs Nic+Coke (P< 0.05); ^#^Nic+Coke vs Nic+Coke+NR (P< 0.05). **Sal+Water vs Nic+Coke (P< 0.01); ##Nic+Coke vs Nic+Coke+NR (P< 0.01).

At the end of the 10 weeks of feeding, mice in the control (Sal+Water), Nic+Water, and Sal+Coke groups gained weight (**Figure 1A**). On the other hand, the Nic+Coke group reduced their body weight by 9.7% compared to Sal+Water group (**Figure 1A**). Nic+S-Coke and Nic+Coke+NR groups lost less body weight from their initial weight (-1.4 and -1.0%, respectively) in relation to Nic+Coke group (-2.3%) (**Figure 1A**).

To elucidate the bodyweight change, we measured the food and liquid intake weekly. The food intake was reduced in the groups drinking S-Coke or Coke (**Figure 1B**); in the Sal plus Coke group, the reduction was 39.3% in relation to Sal plus Water group (1035 vs 629 g). The reduction in the group Nic plus Coke was 13.3% compared to the control group (1035 vs 898 g). A larger decrease (44.2%) in food intake was noted in the group receiving Nic plus Coke in addition to NR (1035 vs 578 g). In the case of the cumulative liquid intake, the groups receiving Coke or S-Coke drank more than the groups without Coke (**Figure 1C**). At the end of the experiment, the group Sal plus Water, and Nic plus Water drank 1250 and 1224 mL, respectively. Meanwhile, the Sal plus Coke and Nic plus S-Coke groups drank 6130 and 6315 mL, respectively. The Nic plus Coke group drank 6263 mL. Interestingly, the addition of NR to Nic plus Coke reduced the volume of Coke drink (6263 vs 5916 mL, respectively). In summary, drinking Coke or S-Coke increased liquid consumption and reduced food intake.

In addition to bodyweight, excessive caloric ingestion is a risk factor to develop NAFLD (40). Considering the food and liquid intake, we calculated the cumulative calories for food, liquid, and total (food plus liquid), as summarized in **Table 1**. **Table 1** shows cumulative food calories, cumulative liquid calories, cumulative liquid consumed, and cumulative total calories (n= 4 or 5 per cage). The groups receiving Nic+S-Coke and Nic+Coke+NR reduced their food caloric intake (2444 and 2360 Kcal, respectively) in relation to Sal plus water group (4224 Kcal). In contrast, the group receiving Nic plus Coke showed a minor reduction of the food calories ingestion in relation to Sal plus Water group (3667 vs 4224 Kcal). Even when mice with Nic plus Coke ingested less food calories than Sal plus Water group, they ingested more food in relation Nic+S-Coke and Nic+Coke+NR groups. When examining liquid calories, only the groups drinking Coke consumed liquid calories, and Nic plus Coke consumed more calories in relation to NR supplemented group (2379 vs 2248 Kcal, respectively). Liquid consumed showed the same pattern as liquid calories analysis. At the end of the experiment, the total calories increased by 14% in the group Nic plus S-Coke, (P< 0,0001), 43% in the group Nic plus Coke (P< 0.0001), and 9% in the group Nic plus Coke and NR (P< 0.001) all compared to the Sal plus Water group. Additionally, the total calories were higher in the Nic plus Coke when we compared to Nic plus S-Coke (P< 0.0001), and Nic plus Coke in the presence of NR reduced the total caloric ingest compared to Nic plus Coke (P< 0.0001). Those results suggest that while Nic plus Coke doesn’t increase body weight, it does increase caloric consumption, however, the addition of NR maintains body weight and caloric consumption.

**Table 1:** Cumulative caloric intake for food, liquid, and total per group.

	Food calories (Kcal)	Liquid calories (Kcal)	Liquid consumed (mL)	Total calories (Kcal)
*Sal+Water*	4224	0	1250	4224
*Nic+Water*	4630	0	1224	4630
*Sal+Coke*	2566	2329	6130	4895
*Nic+S-Coke*	2444**	2399**	6315**	4843**
*Nic+Coke*	3667*^$#^	2379**^#^	6263**^#^	6046**^$#^
*Nic+Coke+NR*	2360**	2248**	5916**	4608*

The calories were calculated in agreement with the grams of food intake or milliliters of liquid drinking week to week. For liquid, the calories have calculated the calories for groups consuming Coke. Total calories (Kcal) were calculated by adding total food and liquid consumption. n= 4 - 5 per group. Statistical difference of the total calorie column is indicated by * compared to Sal+Water (P< 0.001), or ** (P< 0.0001); ^$^Nic+S-Coke vs Nic+Coke (P< 0.0001); ^#^Nic+Coke vs Nic+Coke+NR (P< 0.0001).

### 3.2 Nic plus Coke-induced hepatic steatosis is prevented NR supplementation

**Figure 2:**
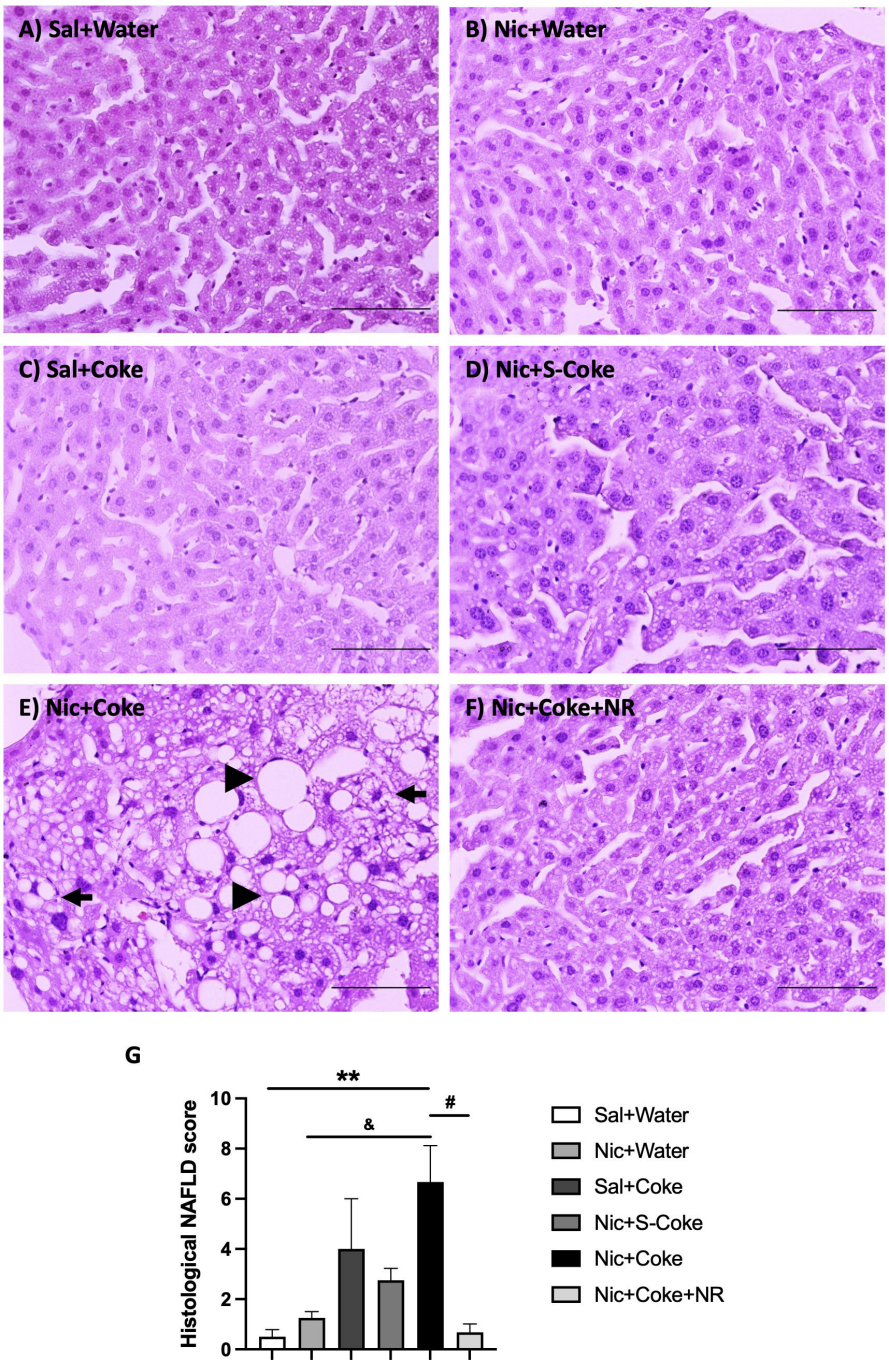
Hepatic steatosis in the Nic+Coke group is reversed by supplementation with NR. Representative light microscopy image of H&E stains liver sections of the mice receiving: **(A)** Sal+Water. **(B)** Nic+Water. **(C)** Sal+Coke. **(D)** Nic+S-Coke. **(E)** Nic+Coke. The arrow indicates a hepatocyte with cytoplasmic microvesicles. The arrowhead shows hepatocyte with cytoplasmic macrovesicles. **(F)** Nic+Coke+NR. Scale bar = 100 *µ*M. **(G)** Quantitative analysis of histological damage of the NAFLD. Values are given as mean ± S.E.M., n= 4 - 5 per group. Statistical difference is indicated by **compared to Sal+Water (P< 0.01); ^&^compared to Nic+ Water (P<0.05); and ^#^compared to Nic+Coke (P< 0.05).

H&E -stained liver sections exhibited normal hepatic structure in mice in the Sal plus Water group, Nic plus Water, Sal plus Coke, and Nic plus S-Coke (**Figures 2A–D**). In contrast, mice received the Nic plus Coke showed lipid accumulation characteristic of both microvesicular and macrovesicular steatosis covering over 66% of the area of the samples (**Figure 2E**, arrow, and arrowhead, respectively), which was prevented by the addition of NR to the Nic plus Coke group (**Figure 2F**). Quantitative analysis (arbitrary units) showed a higher score in Nic plus Coke group (6.6 ± 1.4) in relation to Sal plus Water group (0.5 ± 0.2); the score was increased by the lipid accumulation in the liver cells as microvesicular and macrovesicular steatosis. The score was comparable to Sal plus Water group (0.6 ± 0.3) following the addition of NR (**Figure 2G**). The group receiving S-Coke showed little foci of inflammation with a lower score (2.7 ± 0.4) (**Figure 2G**).

**Figure 3:**
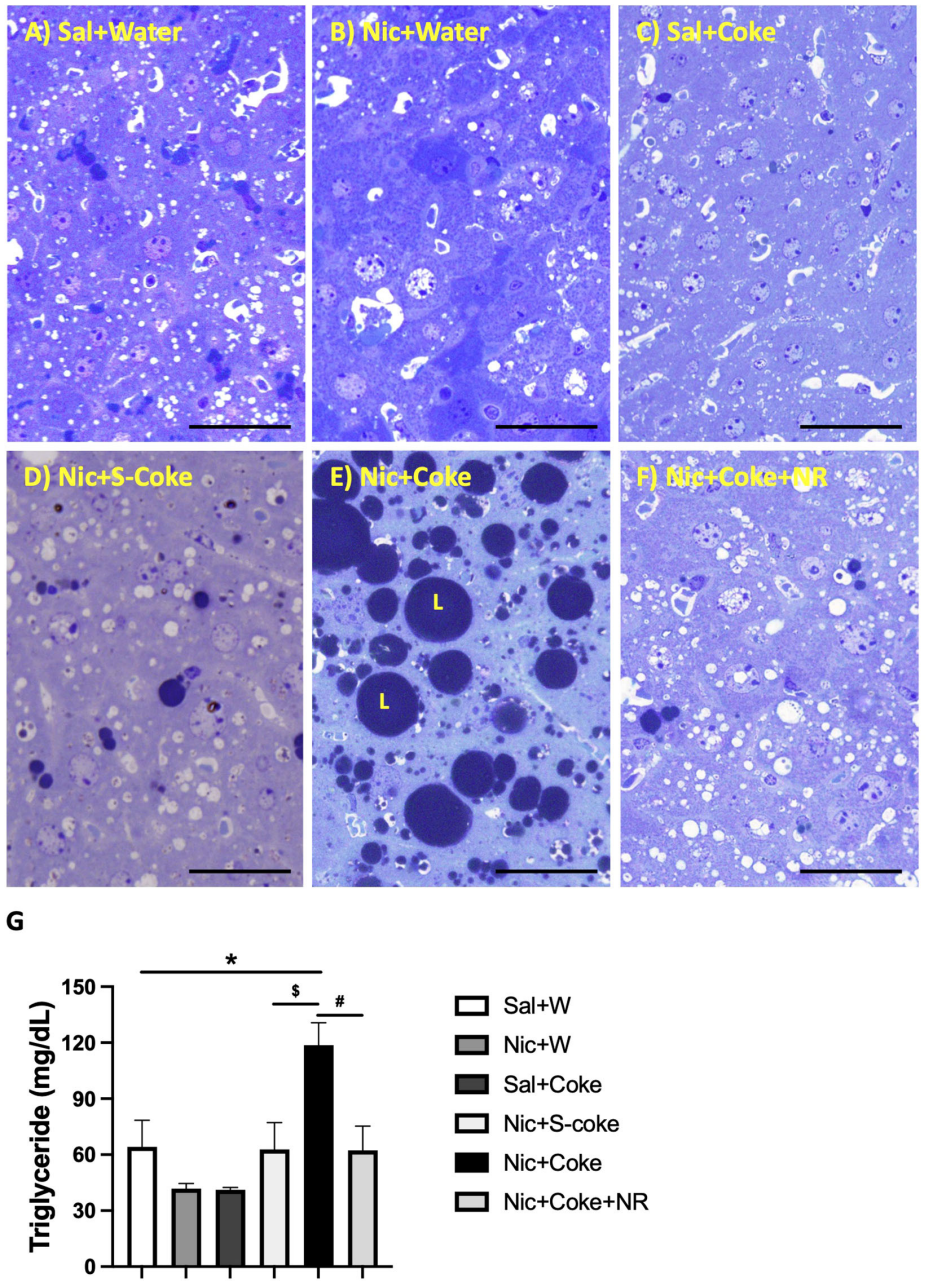
Hepatic lipid accumulation is increased in Nic+Coke group and reversed by NR. Representative high-resolution light microscope images of toluidine blue-stained liver samples of mice fed with **(A)** Sal+Water. **(B)** Nic+Water. **(C)** Sal+Coke. **(D)** Nic+S-Coke. **(E)** Nic+Coke. L= lipid droplet. **(F)** Nic+Coke+NR. Scale bar = 50 *µ*M. **(G)** Quantification of hepatic triglyceride levels. Values are given as mean ± S.E.M., n= 4 - 5 per group. Statistical difference is indicated by *Sal+Water vs Nic+Coke (P< 0.05), ^$^Nic+S-Coke vs Nic+Coke (P< 0.05), ^#^Nic+Coke vs Nic+Coke+NR (P< 0.05).

The above findings were further confirmed by high-resolution light microscopy; using glutaraldehyde-fixed, osmium tetroxide post-fixed, epoxy embedded, and toluidine blue stained liver sections as previously reported (5). A striking increase in larger lipid droplets in Nic plus Coke group (**Figure 3E**) in relation to the other groups, where little or no lipid accumulation were noted (**Figure 3A-F**). As summarized in **Figure 3G**, liver TG content was higher in the Nic plus Coke group (118.7 ± 12 mg/dL) in relation to the Sal+Water (64.1 ± 14.2 mg/dL) and Nic+S-Coke groups (62.8 ± 14.3 mg/dL). In addition of NR to the Nic+Coke group prevented (62.2 ± 12.9 mg/dL) such Nic + Coke induced an increase of the TG in the liver. No significant differences were observed in serum TG levels among various experimental groups (**Supplementary Figure 1A**). However, we observed higher free fatty acid (FFA) levels in the serum of the Nic plus Coke group compared with the Sal plus Water group (8.9 ± 1.5 vs 4.1 ± 1.1 nmol, respectively) (**Supplementary Figure 1B**). The administration of NR together with Nic plus Coke prevented the FFA increase in the serum (1.6 ± 0.6 nmol).

### 3.3 Nic plus Coke increases markers of lipogenesis

**Figure 4:**
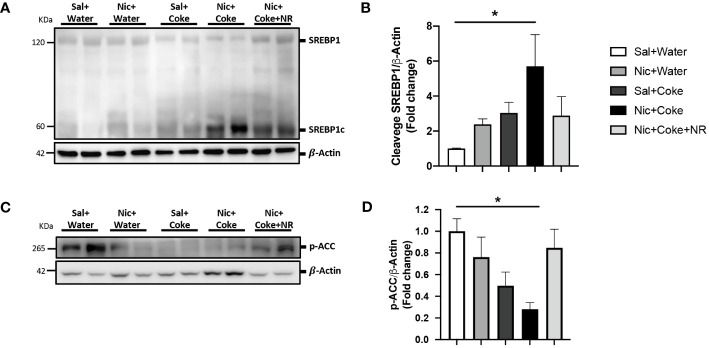
Nic plus Coke administration increases the lipogenesis marker in the liver. **(A)** Representative western blot analysis of SREBP1 cleavage (SREBP1c). β-Actin levels are shown as a loading control. Molecular weight markers are depicted in KDa. **(B)** Quantification of the western blot of SREBP1c. **(C)** Representative western blot analysis of p-ACC. β-Actin levels are shown as a loading control. Molecular weight markers are depicted in KDa. **(D)** Quantification of the western blot of p-ACC. Protein levels of SREBP1c and p-ACC were normalized to β-Actin and expressed as the mean ± S.E.M. (the fold of change relative to the control). n= 5 - 6 per group. Statistical difference is indicated by * compared to Sal+Water vs Nic+Coke (P< 0.05).

Because lipogenesis is activated in NAFLD (41), we next analyzed activation of lipogenesis in Nic + Coke-induced hepatic steatosis. Since we didn’t see any changes in Nic plus S-Coke group compared to the control groups, we focused on HFCS Coke group and their respective control groups. Consistent with lipids deposit, the level of cleaved SREBP1 (SREBP1c) was increased in Nic plus Coke group (**Figures 4A, B**) in relation to the Sal plus Water group. The administration of NR maintained low levels of the SREBP1c, compared to Nic plus Coke group, however, the values were not statistically significant between Nic plus Coke and Nic plus Coke supplemented with NR. To study the transcriptional activity of SREBP1c, we measured the mRNA levels by RT-qPCR analysis of their target genes such as Fatty acid synthase (*FASN*, **Supplementary Figure 2A**) and Stearoyl-CoA 9-desaturase (*Scd1*, **Supplementary Figure 2B**). No significant changes in the expression of *FASN* gene among various treatment groups. However, we observed a significant up-regulation of *Scd1* in the Nic plus Coke in relation to the Sal plus Water group. In addition, we analyzed the phosphorylation of ACC a crucial enzyme for *de novo* synthesis of lipids and NAFLD, which is dephosphorylated to be activated (42). The results showed a reduction of the phosphorylation of ACC (activation signal) (**Figures 4C, D**), suggesting an increase of lipogenesis together with the increase of SREBP1c.

### 3.4 NR administration triggers NAD^+^ signaling decreased by Nic plus Coke

**Figure 5:**
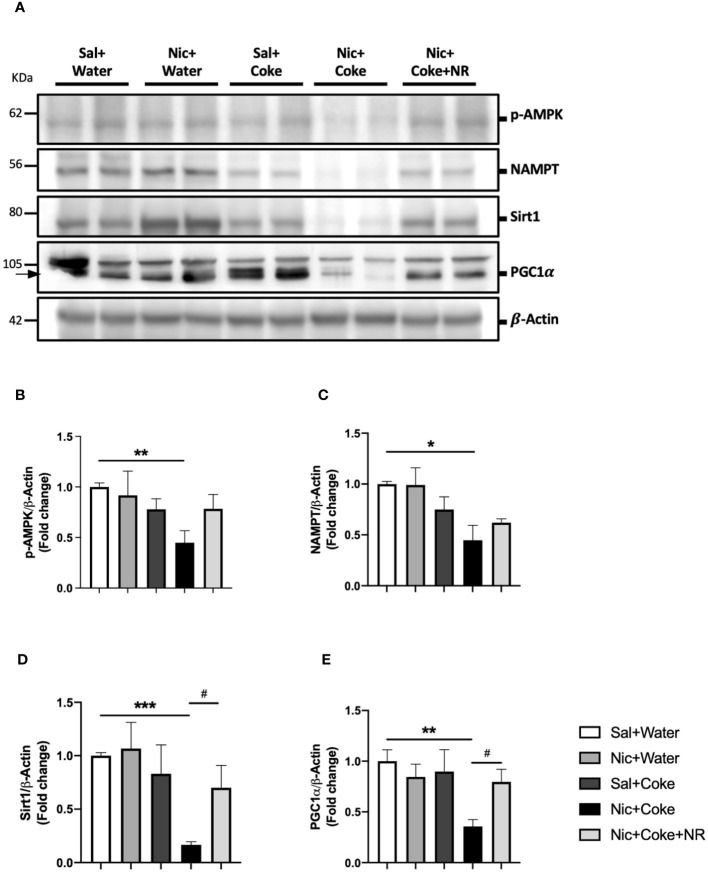
NR administration restores Sirt1 and PGC1*α* signaling pathways reduced by Nic plus Coke-diet. Mice were treated with: Sal+Water, Nic+Water, Sal+Coke, Nic+Coke, and Nic+Coke+NR. **(A)** Representative western blot images for p-AMPK, NAMPT, Sirt1, and PGC1*α*. β-Actin levels are shown as a loading control. Molecular weight markers are depicted in KDa. **(B-E)** Quantitation of band intensities. The protein levels were normalized to β-Actin and expressed as the mean ± S.E.M. (the fold of change relative to the control), n= 5 - 6 per group. Statistical difference is indicated by * compared to Sal+Water (P< 0.05), ** compared to Sal+Water (P< 0.01), *** compared to Sal+Water (P< 0.001), and # compared to Nic+Coke (P< 0.05).

To understand the molecular mechanisms involved in NR-mediated prevention of Nic plus Coke-induced hepatic steatosis, we studied the signaling pathways of NAD^+^ and AMPK, both key regulators of the hepatic metabolism and hepatic steatosis (29, 43, 44). First, we confirmed the NAD^+^ levels in the liver were increased in the NR-receiving group (**Supplementary Figure 3**). Next, we analyzed Nicotinamide phosphoribosyltransferase (NAMPT), which is the enzyme in the first step to produce NAD^+^ (24). The results showed that Nic plus Coke reduced protein levels of NAMPT in relation to Sal plus Water (0.4 ± 0.1-fold relative to Sal plus Water). There were no significant differences in NAMPT levels among the other groups (**Figures 5A, C**). The NAD^+^ produced by the enzyme NAMPT promotes the activity of Sirt1, a downstream target of NAD^+^, to regulate lipid homeostasis through increased expression of PGC1*α* (30, 45). Consistent with this, Nic plus Coke decreased the protein levels of Sirt1 (0.1 ± 0.02-fold relative to Sal plus Water) (**Figures 5A, D**) and PGC1*α* (0.3 ± 0.02-fold relative to Sal plus Water) (**Figures 5A, E**), which were restored by NR for Sirt1 (0.7 ± 0.2-fold relative to Sal plus Water), and PGC1*α* (0.8 ± 0.02-fold relative to Sal plus Water). Because AMPK regulates lipid metabolism, we next analyzed the phosphorylation status of AMPK. We found a significant decrease in p-AMPK in the Nic plus Coke group (0.4 ± 0.1-fold relative to Sal plus Water), which was partially, but significantly restored by NR treatment (0.7 ± 0.1-fold relative to Sal plus Water) (**Figures 5A, B**).

### 3.5 NR reduces oxidative stress and prevents mitochondrial abnormalities.

**Figure 6:**
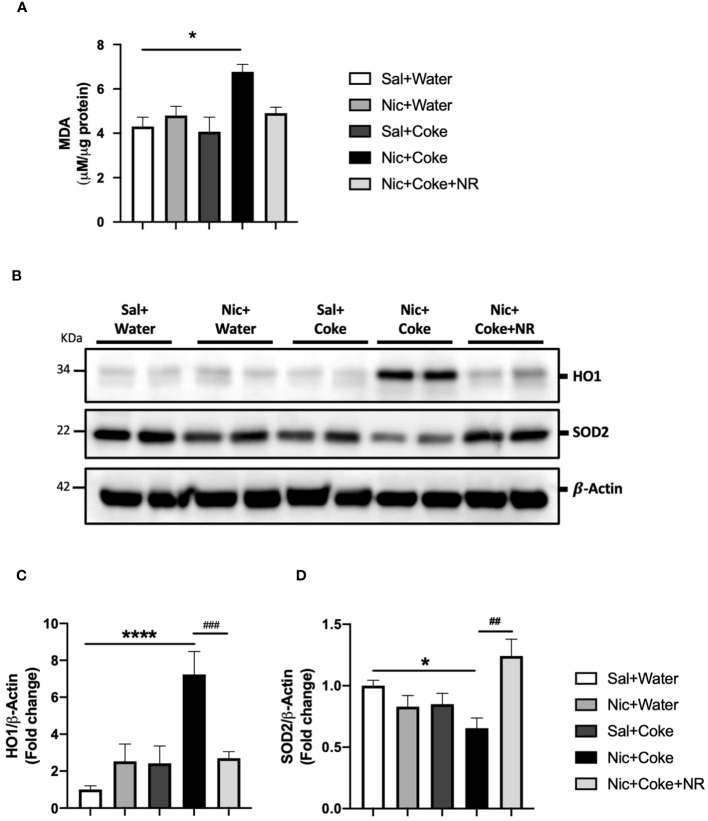
NR reduces oxidative stress increased by Nic plus Coke. Mice were treated with: Sal+Water, Nic+Water, Sal+Coke, Nic+Coke, and Nic+Coke+NR. **(A)** Lipid peroxidation measured by MDA quantification in liver samples. **(B)** Representative western blot images of HO1 and SOD2. β-Actin levels are shown as a loading control. Molecular weight markers are depicted in KDa. The quantitative analysis of the western blot is shown for HO1 in **(C)** and SOD2 in **(D)**. The protein levels were normalized to β-Actin and expressed as the mean ± S.E.M. (the fold of change relative to the control), n= 5 - 6 per group. Statistical difference is indicated by * compared to Sal+Water (P< 0.05), **** compared to Sal+Water (P< 0.0001), # compared to Nic+Coke (P< 0.05), and ## compared to Nic+Coke (P< 0.01).

Given that oxidative stress is an active contributor to the development of hepatic steatosis (46), we analyzed lipid peroxidation through MDA formation. Nic plus Coke group had increased MDA levels in comparison with the Sal plus Water group (6.7 ± 0.3 vs 4.3 ± 0.4, respectively) (**Figure 6A**). To complement our results, we carried out western blot analysis of Heme oxygenase 1 (HO1) and Superoxide Dismutase 2 (SOD2). HO1 is activated in response to the increase of the reactive oxygen species (ROS) to reduce them, and SOD2 is reduced when ROS are increased. Our results showed that Nic plus Coke increased hepatic HO1 levels (7.2 ± 1.2-fold) relative to Sal plus Water group (**Figures 6B, C**) coupled with a reduction in SOD2 levels (0.6 ± 0.08-fold) relative to Sal plus Water group (**Figures 6B, D**). NR administration decreased HO1 protein levels (2.6 ± 0.3-fold) and increased SOD2 protein levels (1.2 ± 0.1-fold relative to Sal plus Water group. These results suggest that Nic plus Coke produces higher oxidative stress, which can be mitigated by NR supplementation.

**Figure 7:**
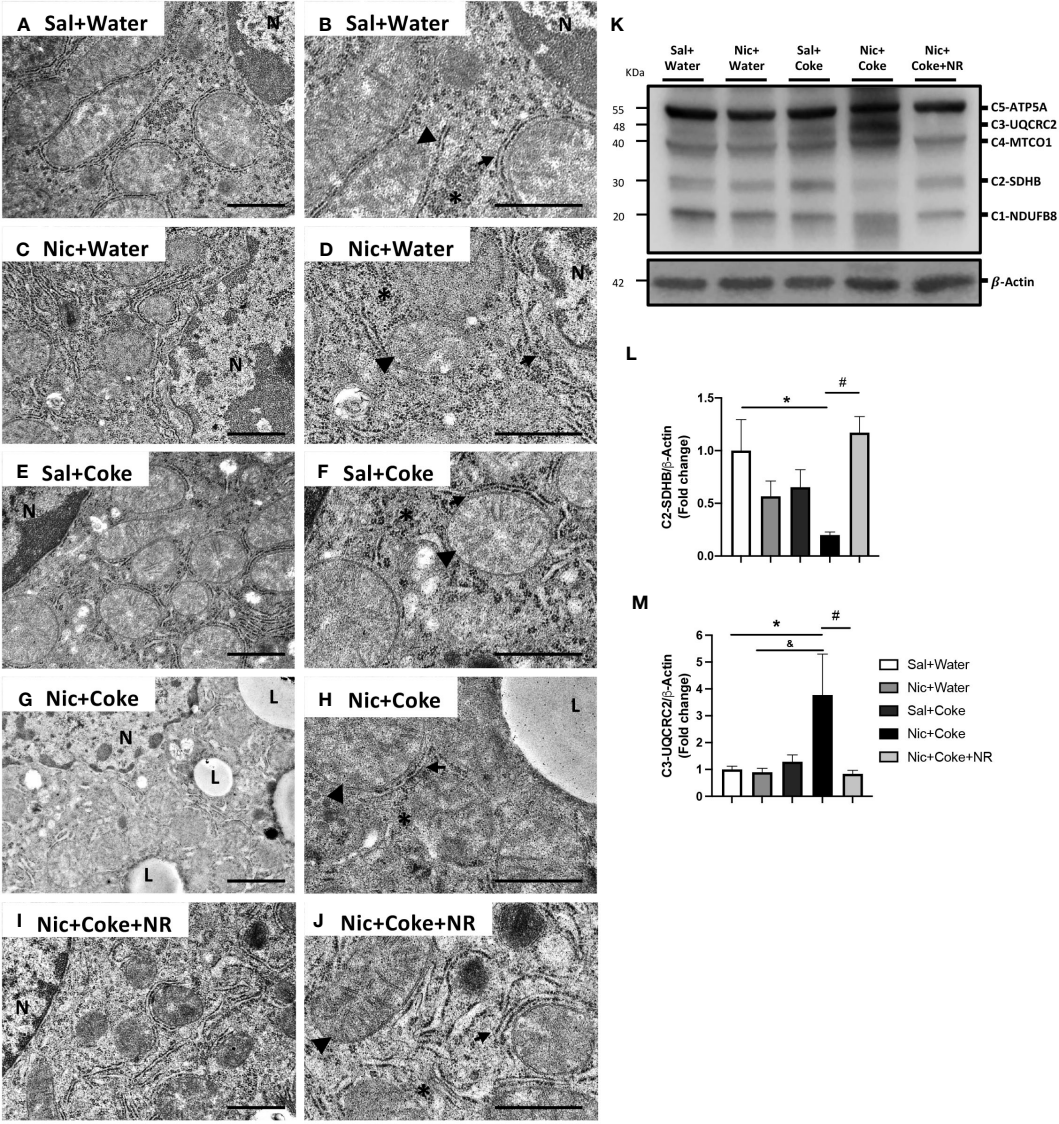
Mitochondrial ultrastructural damage induced by Nic+Coke is prevented by NR supplementation. Representative transmission electron microscopic images of lower magnification of the groups **(A)** Sal+Water. **(C)** Nic+Water. **(E)** Sal+Coke. **(G)** Nic+Coke, and **(I)** Nic+Coke+NR. **(B, D, F, H, J)** higher magnification of **(A, C, E, G, I)**, respectively. N= nucleus, L= lipid droplet. In all images, the arrowhead is the mitochondria, the arrow indicates the endoplasmic reticulum, and the asterisk is the glycogen. Scale bar for lower magnification= 1 *µ*m, and higher magnification= 800 nm. In addition, the liver was excised and homogenized to evaluate electron transporter chain (ETC) proteins from complex 1 to 5 by western blot. **(K)** Representative western blot image of ETC. β-Actin levels are shown as a loading control. Molecular weight markers are depicted in KDa. The quantitative analysis of the western blot is shown for Complex 2 in **(L)** and Complex 3 in **(M)**. The protein levels were normalized to β-Actin and expressed as the mean ± S.E.M. (the fold of change relative to the control), n= 5 - 6 per group. Statistical difference is indicated by * compared to Sal+Water (P< 0.05), ^&^ compared to Nic+ Water (P<0.05), and ^#^ compared to Nic+Coke (P< 0.05).

Next, we analyzed the mitochondrial changes, since they are the principal source of generating oxidative stress, and Nic can also generate hepatic oxidative stress (5, 34). We performed electron microscopy to assess cyto-architecture of mitochondria in various treatment groups. Hepatocytes from Sal plus Water (**Figures 7A, B**), Nic alone (**Figures 7C, D**), or Coke alone groups (**Figures 7E, F**), showed normal architecture characterized by numerous mitochondria with well-defined mitochondrial cristae (head arrow), smooth- and rough endoplasmic reticulum (arrow), and glycogen deposition (yellow asterisks) with minimal lipid accumulation (**Figures 7A, B**). Notably, the addition of Nic to Coke led to a striking increase in lipid accumulation (L) along with mitochondrial swelling and loss of the mitochondrial cristae (arrowhead), with a decrease in the amount of endoplasmic reticulum (arrow) and glycogen deposition (yellow asterisk) (**Figures 7G, H**). The addition of NR to Coke was able to prevent these changes (**Figures 7I, J**).

To further explore the mitochondrial damage, we evaluated the protein levels of the electron transport chain (ETC) subunits by western blotting (**Figure 7K**). We studied complex-1 (C1) NADH : Ubiquinone Oxidoreductase Subunit B8 (NUDFB8), C2 Succinate Dehydrogenase Complex Iron-Sulfur Subunit B (SDHB), C3 Ubiquinol-Cytochrome C Reductase Core Protein 2 (UQCRC2), C4 Mitochondrial Encoded Cytochrome C Oxidase I (MTCO1), and C5 ATP synthase F1 subunit alpha (ATP5A) of ETC. The analysis showed that Nic plus Coke reduced the C2-SDHB (0.2 ± 0.02-fold relative to Sal plus Water), which was fully attenuated (1.1 ± 0.1-fold relative to Sal plus Water) by NR (**Figures 7K, L**). Likewise, levels of C3-UQCRC2 also increased with the administration of Nic and Coke (3.7 ± 1.5-fold relative to Sal plus Water) but were attenuated by NR (0.8 ± 0.1-fold relative to Sal plus Water) (**Figures 7K, M**). No significant differences were noted in the levels of C1-NUDFB8, C4-MTCO1, and C5-ATP5A in various experimental groups (**Supplementary Figure 4**). Together, these results indicate that administration of NR reduced the markers of oxidative stress and prevented the mitochondrial damages induced by Nic and Coke.

## 4 Discussion

In this study, we showed that Nic or HFCS (Coke) alone doesn’t lead to hepatic steatosis with a normal chow diet even though cigarette smoking and HFCS are linked to metabolic diseases such as NAFLD (4, 18, 41, 47). However, the combination of Nic and HFCS (Coke) has an additive effect leading to hepatic steatosis (**Figure 2**). In an effort to uncover mechanisms of the etiology of NAFLD that could lead to an effective therapy for this condition, we propose that supplementation with NR that prevents liver damage (27, 29, 45) and increases NAD^+^ signaling (48), could be useful for preventing hepatic steatosis induced by agents such as Nic in combination with Coke with HFCS or a HFD. Furthermore, NAFLD can be caused by a HFD together with a reduced NAD^+^ concentration as is present in mice with the genetic disruption of NAMPT (29, 49).

NAD^+^ is irreversibly degraded by enzymes such as sirtuins, so external supplementation to maintain cellular concentrations of NAD^+^ is important to avoid diseases such as NAFLD. In our study, supplementation with the precursor of NAD^+^, NR, prevented hepatic steatosis induced by Nic plus HFCS (Coke), involving specific molecular signaling downstream of NAD^+^, Sirt1, and PGC1*α*, to reverse mitochondrial damage. NAD^+^ is a pivotal metabolite to liver function, and these results highlight the central role of NAD^+^ as a treatment target and consistent with other results found in hepatic steatosis (25, 50, 51).

Previously, our group showed that Nic plus a second hit such as HFD produces hepatic steatosis (5). In the current study, we observed similar consequences with Nic, but now our second hit was HFCS from the SSBs (Coke). One of the principal consequences and important to the diagnosis of NAFLD is the changes in the hepatic tissue presented as a distortion of the morphology characterized by lipid deposits in more than 5% of the hepatic cells (46, 52), that we observed with H&E staining, and confirmed the lipid deposition with toluidine blue staining, only in the Nic plus Coke group (**Figures 1**, **2**, respectively). Interestingly, we incorporated a Nic plus sugar-sucrose (cane sugar) group to investigate whether sucrose from SSBs (S-Coke) induced the same effects as HFCS SSBs (Coke) (**Figures 1**, **2**). However, Nic plus S-Coke didn’t produce hepatic steatosis in relation to Nic plus Coke (HFCS), as the Nic plus S-Coke group had only few lipid droplets deposit in relation to Nic plus Coke.

Why does Nic plus sucrose-sweetened Coke not lead to hepatic steatosis while Nic plus HFCS Coke does? A possible explanation was a study by Zhao et al. that showed that fructose is transformed into acetate by gut microbiota so that hepatocytes produce acetyl-CoA and convert it into lipids (53). Alternatively, high consumption of fructose increased its availability in the hepatocytes augmenting the intermediate citrate which is broken to generate 2 molecules of acetyl-CoA to enhance lipogenesis (53).

Glucose (sucrose) and fructose (HFCS) have different metabolism, which also can explain the different outcomes of drinking S-Coke (sucrose) or Coke (HFCS) (54). Moreover, a study in Juvenile Iberian pigs reported that HFCS has worst consequences than sucrose in increasing adipose tissue and serum triglycerides (55), and fructose can increase fatty acid synthesis in the liver of human volunteers (21). We observed an increase in the lipogenic transcription factor SREBP1 cleavage (activation) and dephosphorylation of ACC (activation), together with increased lipid droplets and TGs demonstrating the increased fat accumulation in the liver (**Figures 2**, **3**). ACC is a target of AMPK and the dephosphorylation of ACC is supported by the reduction of p-AMPK as we reported (56). Previously, it was described that fructose stimulated *de novo* lipogenesis by the activation of SREBP1 (57, 58), and SREBP1 promoted transcriptional activity to increase lipogenic enzymes such as ACC, *FASN*, or *Scd1* (59). Our transcriptional analysis of the RNA levels of *FASN* and *Scd1* showed an increase only in *Scd1* but not in *FASN* (**Supplementary Figure 2**). Though both genes are targets of SREBP1c, it is known that *FASN* is responsive to a high glucose diet (60) and *Scd1* has a higher transcriptional response to a high fructose diet (61, 62). However, we cannot rule out the possibility of an SREBP1c-independent mechanism in the transcriptional regulation of *Scd1* (61). We also cannot rule out possibility that other transcription factors closely related to SREBP1c such as the carbohydrate response element binding protein (ChREBP) or LXR that are associated with lipogenesis and NAFLD (63, 64) may also have contributed to promote the lipogenic machinery. This clearly merits further investigation.

The increase in lipogenesis markers and lipid deposits by Nic and Coke was correlated with a reduction of opposing signaling pathways related to NAD^+^, Sirt1, and PGC1*α* (**Figure 5**). Conditions such as aging and the reduction of the NAD^+^ concentration lead to decreased Sirt1 and PGC1*α* signaling (27, 65–67), which is important as PGC1*α* is the downstream target of Sirt1. The reduction of Sirt1, and PGC1*α* could be correlated with a decrease in the NAD^+^ salvage pathway (NAMPT) and mitochondrial damage which is supported by the reduction of the phosphorylation of AMPK (**Figure 5**). Supplementation with NR in the presence of Nic plus Coke leads to an increase in Sirt1 and PGC1*α* likely due to increased NAD^+^ concentration (**Supplementary Figure 3**) in agreement with previous publications that showed NR administration increased liver-mitochondrial NAD^+^ concentration (22, 32, 51). However, we didn’t obtain a recovery of the AMPK activity after NR treatment (as seen in **Figures 5A, B**). AMPK and NAD^+^ signaling are closely related, as AMPK stimulates NAD^+^ downstream signaling, such as Sirt1 to enhance NAD^+^ (32, 68). The mechanism of action of the NR doesn’t involve AMPK, because NR is a precursor of nicotinamide mononucleotide (NMN) through the salvage pathway to form NAD^+^ (69), and NR doesn’t involve AMPK interaction.

Mitochondrial damage together with oxidative stress are pivotal mechanisms of NAFLD development (46, 70), as we found with Nic plus Coke group. In the mitochondria, the ETC proteins were altered with the decrease of C2-SDHB and increase of the C3-UQCRC2 (**Figure 7**). C2-SDHB is an acceptor of the electrons from lipid oxidation (71). However, as the mice are exposed to a carbohydrate (HFCS) source, the flux to lipid degradation in the liver is expected to decrease, even when the lipid synthesis is increasing in the Nic plus Coke group. This is correlated with our experimental conditions, where the group receiving Nic plus Coke has an increased consumption of carbohydrates from Coke and reduction of the food intake (normal chow) (**Figure 1**). On the other hand, the increase of the C3-UQCRC2 has been shown to increase oxidative stress production from mitochondria (72). In our results, the increase of the C3-UQCRC2 is correlated with the increase of the lipid peroxidation (MDA) and the antioxidant and responsive enzyme HO1, which is a hepatoprotective enzyme (73, 74). In addition, the antioxidant enzyme SOD2, localized in the mitochondrial matrix (75), is reduced and we found ultrastructural mitochondrial damage with fewer crests and swelling in the Nic plus Coke group. Thus, our results strongly suggest increased hepatic oxidative stress and mitochondrial damage, which can be prevented by NR supplementation. We could explain this prevention by the increase of the Sirt1 and PGC1*α*, which might promote mitochondrial biogenesis (32). Other studies also reported a protective role of NR in the mitochondria (76).

We showed the NAD^+^ increased with NR treatment on hepatocytes (**Supplementary Figure 3**). Other articles provided evidence that systemic administration of NR at the same doses used by us (400 mg/kg) increased NAD^+^ in the liver tissue without involving the salvage pathway and specifically the NAD^+^ pool in the mitochondria of the liver (32). This is consistent with our findings because we also didn’t observe the recovery of the protein levels of the enzyme NAMPT (**Figures 5A, C**), which participate in the salvage pathway. In addition, we showed an improvement in the mitochondrial structure and ETC proteins in the group with NR supplementation (**Figure 7**) supported by the high levels of NAD^+^ (**Supplementary Figure 3**). Additionally, we performed experiments with Water plus NR treatment and did not find changes in the body weight, liquid or food consumption, calorie intake, and histology in comparison to the Saline plus Water group (unpublished data). This is consistent with previous results that under a normal chow diet, NR does not affect parameters such as body weight or food consumption (32).

We further believe that the results are direct consequence of NR on the liver tissue since only hepatic TG levels (**Figure 3G**) not serum TG levels (**Supplementary Figure 1A**) are reduced in the group supplemented with NR (**Figure 3G**), suggesting that the NR effect doesn’t involve the release of the TG from adipose tissue.

Previous studies have shown that Nic administration in addition to HFD produces hepatic steatosis and the prevalence of NAFLD has a direct relation to the number of cigarettes smoked per day (77). As a multi-hit disease, NAFLD risk increases with the addition of other factors (4), and we analyzed two of them Nic and HFCS which had not been previously studied together. The groups drinking Coke (S-Coke or HFCS Coke) drank more Coke than water increasing drinking volume and caloric ingest, and at the same time these groups reduced the quantity of food intake (**Figure 1**; **Table 1**), so the caloric ingestion was higher than the groups without Coke. However, among the Coke groups the Nic plus Coke group consumed 30% more food than the Nic plus S-Coke, and the addition of NR to Nic plus Coke maintained the food consumption similar to Nic plus S-Coke group (**Figure 1**; **Table 1**). The 30% higher food intake of the Nic plus Coke group produces an excess of calories. This is an important finding because calories are one of the key triggers of NAFLD, patients with NAFLD consume an excess of calories and caloric restriction is an effective therapy (78, 79). In this context, HFCS can lead to an increase in carbohydrates and a reduction in protein intake, together with a reduction in the quality of the food (80). The increase in caloric ingestion could be explained because fructose, unlike sucrose, doesn’t stimulate the release of leptin or insulin, and lipolysis is blocked, reducing satiety and contributing to increased food consumption and lipid storage in the hepatic cells of the fructose (81, 82). Interestingly, the restriction of fructose in the diet reduced hepatic lipid deposit and *de novo* lipogenesis (83), while a hypercaloric diet with fructose increased lipid liver deposits (84). In contrast, in young health people, high consumption of fructose in an isocaloric diet did not enough to produce changes in the liver (85), but an isocaloric diet of fructose or glucose beverages plus a second hit such as HFD triggers metabolic detrimental effects only with fructose. In this context, Nic increases the lipolysis from adipose tissue and increases the lipids accumulation in the liver (5), explaining the Nic contribution to higher hepatic damage in the Nic plus Coke group. As mentioned, the reduction in calorie intake in the group receiving NR is an important finding. Another work showed that an increase in NAD^+^ produces a reduction of food intake (86). Although mechanisms of caloric intake are complex, it is suggested that NAD^+^ controls in the hypothalamus by the regulation of the cellular clock machinery recovering the metabolic behaviors (86). Interestingly, the overexpression of Sirt1 at hypothalamus neurons suppressed food intake (87). In our results, Sirt1 is recovered by NR supplementation (**Figures 5A, D**), and the treatment with NR is systemic, so we can speculate that Sirt1 could be increased at the hypothalamus by NAD^+^ in the NR supplemented group to reduce the calorie intake. However, this merits further investigation.

Even with this high calorie intake in Coke groups, they don’t increase the body weight which is supported by the weight loss effect of Nic as our group published previously (88). It’s widely known of the paradoxical effect of Nic that leads to metabolic diseases in spite of weight reduction (36, 89, 90), and it has been suggested that Nic increased fat metabolism avoiding weight gain (91). Importantly, non-overweight patients consuming fructose still develop NAFLD (92); thus redistribution of adipose tissue likely plays a key role in the development of NAFLD.

The sweetener HFCS is a health concern problem because it is associated with NAFLD with and without metabolic symptoms (93). The higher consumers of SSBs are youths (especially of color) influenced by attractive marketing (12, 94). On the other hand, new ways to deliver Nic have been introduced in the market as electronic cigarettes that lead to hepatic steatosis (34), and are extremely popular among youth, with attractive marketing offering different technological formats and flavors, such as vanilla are by themselves toxic for hepatic cells after *in vitro* exposure (95). Moreover, reports of consumers of electronic cigarette products with lung injury (EVALI) showed an increase in plasma liver transaminases enzymes (96, 97). This suggests that the new devices could produce more severe hepatic damage together SSBs, and the recent trend of adolescents vaping as well as consuming SSBs is concerning, thus our studies are timely.

**Figure 8:**
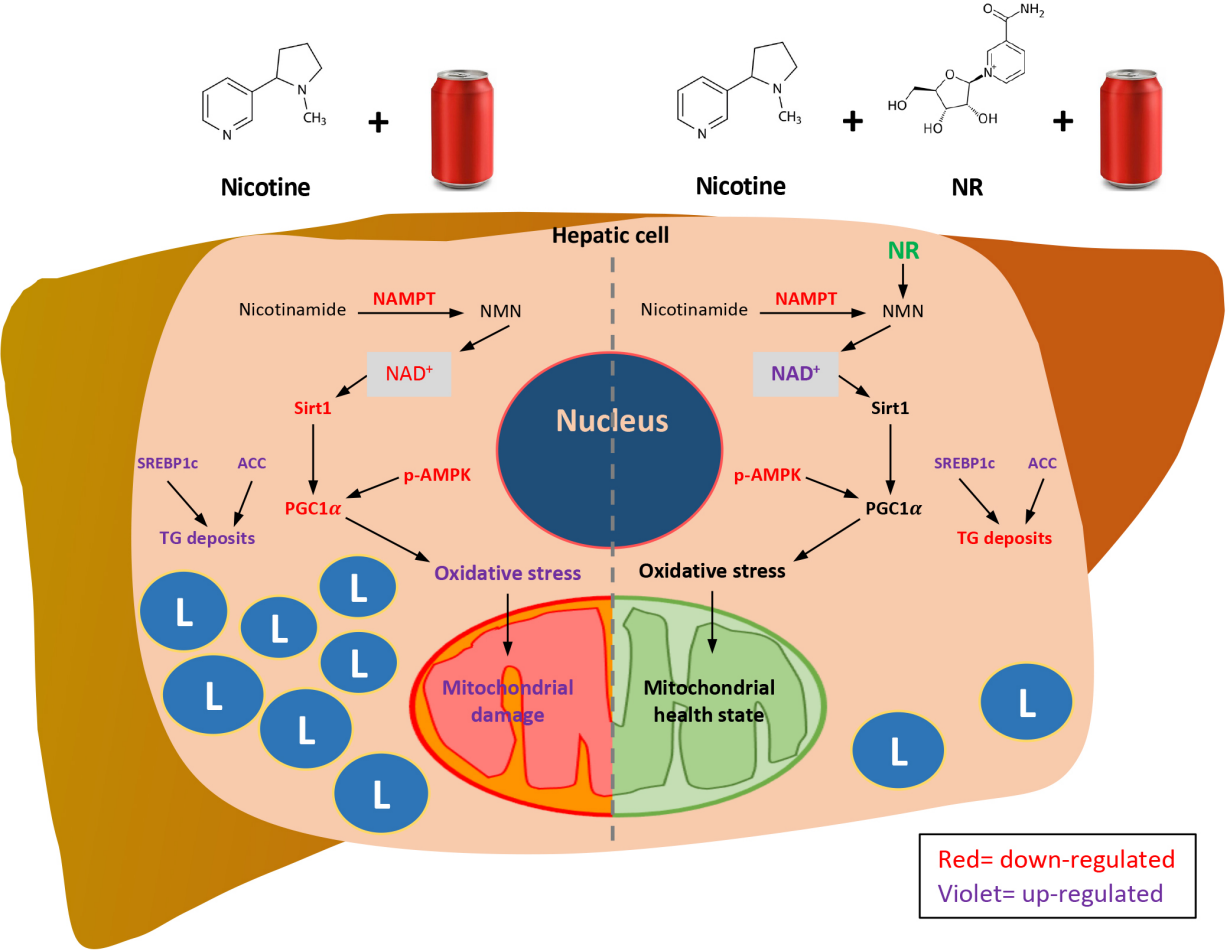
Proposed mechanisms of how NR protects Nic plus Coke-induced hepatic steatosis, and the protective effect of the NR supplementation. L=TG deposits. Red letters indicate down-regulation. Violet letters indicate up-regulation.

We propose (**Figure 8**) that Nic plus Coke consumption reduced the protein levels of the NAMPT, and decreased the NAD^+^-dependent enzyme, Sirt1. Downstream of Sirt1, PGC1α was also reduced. In addition, Nic plus Coke promotes SREBP1c and ACC (active forms) and intracellular TG deposits. The phosphorylation of AMPK was also reduced. PGC1α reduction could maintain old and damaged mitochondria with high oxidative stress (MDA, HO1 increase, and SOD2 decrease). Moreover, incorporating NR into the diet increased NAD^+^ and protein levels of Sirt1 and PGC1α, that would promote a healthy mitochondrial state with reduced oxidative stress and recovering mitochondrial proteins. NAMPT and p-AMPK reduction, and SREBP1c increase were unchanged after NR treatment. Lipid deposits were decreased probably due to the recovery of mitochondria. This proposed model may be complemented in the future to determine key mechanisms such as lipogenesis, mitochondrial biogenesis, and mitochondrial function.

In summary, we found the additive effect of the Nic plus Coke administration to cause hepatic steatosis in mice as a NAFLD model, as well as the protection of the NR supplementation (**Figure 8**). Our results suggest that NR leads to a decrease in the calorie intake to reduce hepatic steatosis through the increased of the NAD^+^ concentration, and triggers the downstream signaling of NAD^+^, with a reduction in oxidative stress markers and mitochondrial damage.

## Data availability statement

Publicly available datasets were analyzed in this study. This data can be found here: Dryad, https://datadryad.org/stash, DOI: 10.5061/dryad.g1jwstqx7.

## Ethics statement

The animal study was approved by Charles R. Drew University and Lundquist Institute Animal Care and Use Committees (IACUC). The study was conducted in accordance with the local legislation and institutional requirements.

## Author contributions

JR: Writing – original draft, Methodology, Formal analysis, Data curation. JE-D: Writing – review & editing, Supervision, Investigation, Conceptualization. KMH: Writing – review & editing, Supervision, Investigation, Conceptualization. JM-M: Writing – review & editing, Investigation. JM: Writing – review & editing, Investigation. CJL: Writing – review & editing, Investigation. ML: Writing – review & editing, Software, Validation, Formal analysis, Data curation. DL: Writing – review & editing, Investigation. JW: Writing – review & editing, Investigation. AS-H: Writing – review & editing, Validation, Supervision, Conceptualization. TF: Writing – review & editing, Validation, Supervision, Resources, Funding acquisition, Conceptualization.

## Supplementary material

Supplementary Figure 1 Serum triglyceride and FFA levels. Blood samples were collected to obtain the serum as previously reported (5). **(A)** Samples were processed and quantified according to the manufacturer’s instruction to measure triglycerides with the Triglyceride Colorimetric Assay Kit (Cayman Chemical, MI, USA). **(B)** FFAs were quantified using the commercial kit Free Fatty Acid Assay Kit following the manufacturer instructions (Abcam, CA, USA). The graph is expressed as the mean ± S.E.M. n= 5 - 6 per group. Statistical difference is indicated by * compared to Sal+Water (P< 0.05) and ^##^ compared to Nic+Coke (P< 0.01).

Supplementary Figure 2 Transcriptional analysis of lipogenesis genes. Total RNA was extracted from the experimental groups using TRIzol reagent (Invitrogen) and a higher 260/280 ratio was quantified in the NanoDrop 2000 (Thermo Fisher Scientific) as previously reported (39). Briefly, pure RNA (1 ug) was reverse transcripted to cDNA using OligodT and the High Quality RNA-to-cDNA kit (Applied Biosystems). Quantitative Real-Time PCR (qPCR) was done in duplicates, using the Step-One plus RT-PCR system (Life Technology) and the SYBR Green PCR Master Mix (Applied Biosystems). The primers were the following: *18S*: (F) 5’-GTAACCCGTTGAACCCCATT-3’ and (R) 5’-CCATCCAATCGGTAGTAGCG-3’; *Fasn*: 5’-GCTGCGAAACTTCAGGAAAT-3’ and (R) 5’-AGAGACGTGTCACTCCTGGACTT-3’; *Scd1*: 5’-CCTTCCCCTTCGACTACTCTG-3’ and (R) 5’-GCCATGCAGTCGATGAAGAA-3’. The analysis was normalized to the housekeeping gene *18S* and we quantified the RNA expression using the 2^-ΔΔCT^ method for relative quantification. The quantitative analysis is shown in graphs **(A)** *Fasn* and **(B)** *Scd1*. The graphs are expressed as mean ± S.E.M. (the fold of change relative to the control), n= 4 - 5 per group. The statistical difference is indicated by * compared to Sal+Water (P< 0.05). *Fasn*: fatty acid synthase and *Scd1*: Stearoyl-CoA 9-desaturase.

Supplementary Figure 3 NR supplementation increases the NAD^+^ concentration in the liver. NAD^+^ levels were analyzed using the commercial EnzyChrom™ NAD/NADH Assay kit (BioAssay Systems, CA, USA) according to manufacturer protocol. NAD^+^ levels were normalized by liver weight in grams. The graph is expressed as the mean ± S.E.M. n= 3 - 5 per group. The statistical difference is indicated by ^#^ compared to Nic+Coke (P< 0.05).

Supplementary Figure 4 Electron transporter protein levels. The liver was excised and homogenized to evaluate electron transporter chain (ETC) proteins from complex 1 to 5 by western blot. Representative western blot image of ETC. β-Actin levels are shown as a loading control. Molecular weight markers are depicted in KDa. The quantitative analysis of the western blot is shown for Complex 1 to 5. The protein levels were normalized to β-Actin and expressed as the mean ± S.E.M. (the fold of change relative to the control), n= 5 - 6 per group. Statistical difference is indicated by * compared to Sal+Water (P< 0.05), ^&^ compared to Nic+ Water (P<0.05), and ^#^ compared to Nic+Coke (P< 0.05).

Supplementary Table 1 Treatment injection schedule. Each injection was 200 μl.

Supplementary Table 2 Scoring for rodent NAFLD (35).
