# Supplementary figures and images for: Hepatic steatosis induced by nicotine plus Coca-Cola™ is prevented by nicotinamide riboside (NR)
Source: Front Endocrinol (Lausanne). 2024 May 2;15:1282231. doi: 10.3389/fendo.2024.1282231 (PMC11097688; doi:10.3389/fendo.2024.1282231)

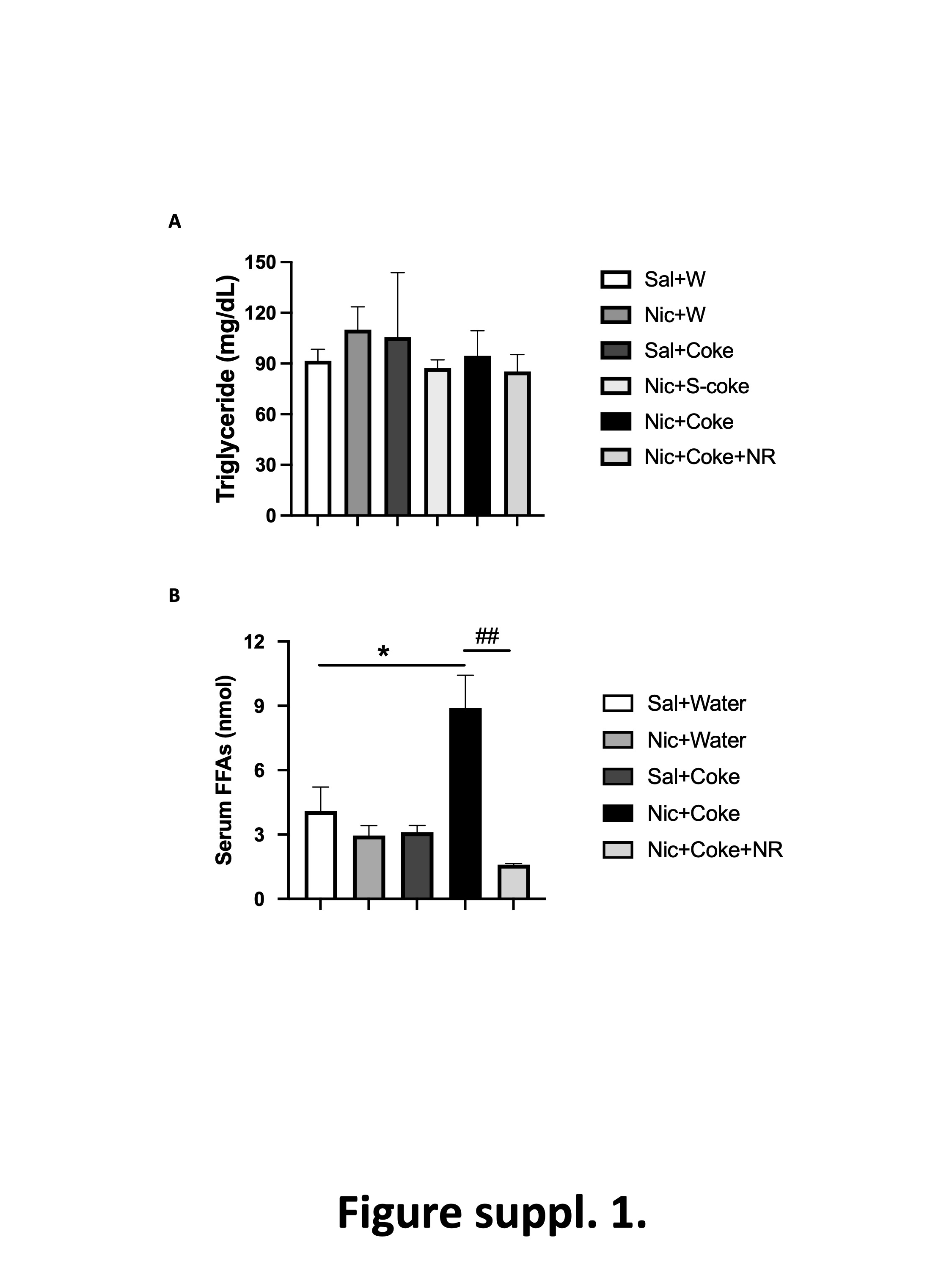

Supplement: Supplementary Figure 1 — Serum triglyceride and FFA levels. Blood samples were collected to obtain the serum as previously reported (5). (A) Samples were processed and quantified according to the manufacturer’s instruction to measure triglycerides with the Triglyceride Colorimetric Assay Kit (Cayman Chemical, MI, USA). (B) FFAs were quantified using the commercial kit Free Fatty Acid Assay Kit following the manufacturer instructions (Abcam, CA, USA). The graph is expressed as the mean ± S.E.M. n= 5 - 6 per group. Statistical difference is indicated by * compared to Sal+Water (P< 0.05) and ## compared to Nic+Coke (P< 0.01). [file Image_1.jpeg]

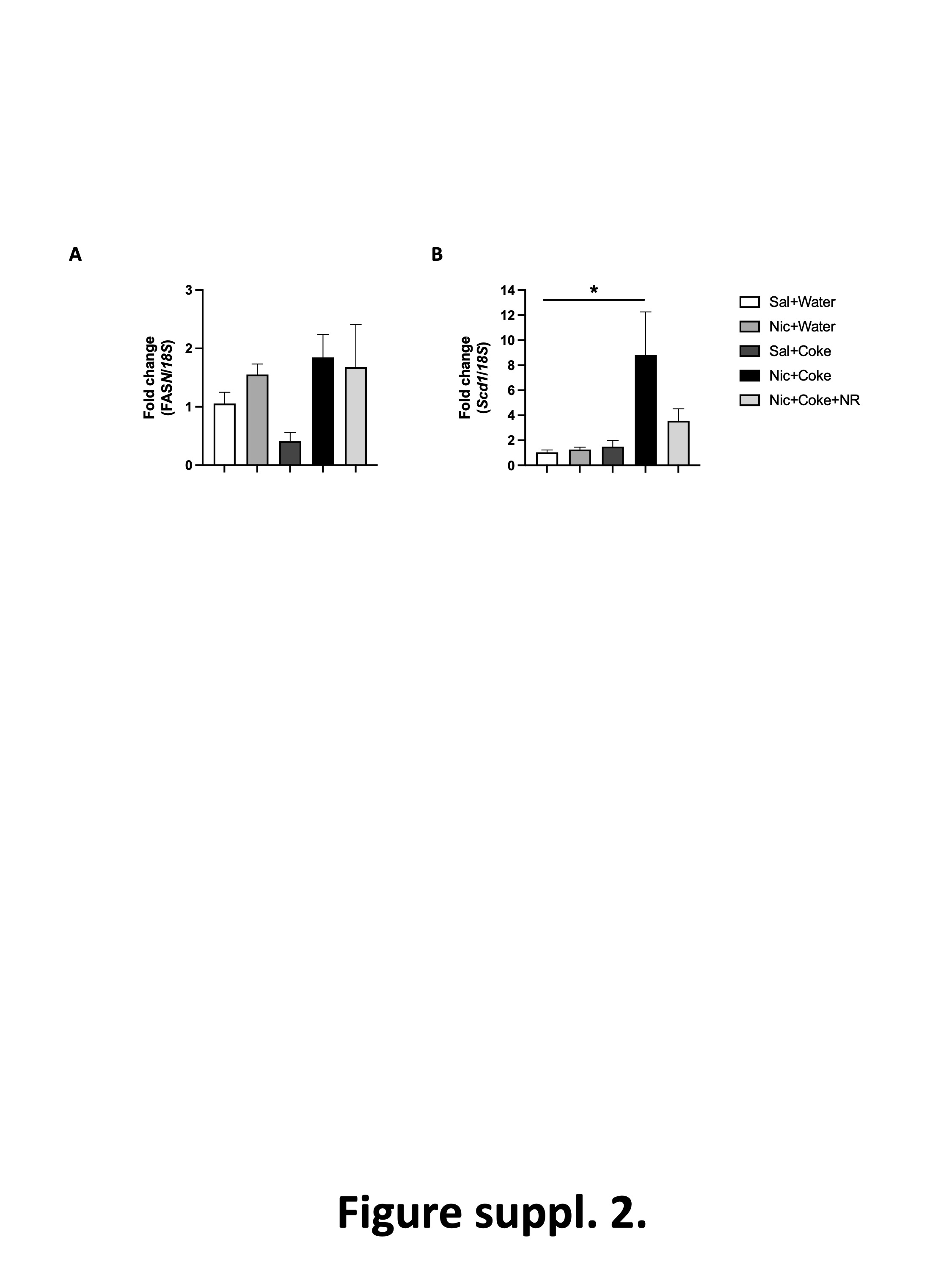

Supplement: Supplementary Figure 2 — Transcriptional analysis of lipogenesis genes. Total RNA was extracted from the experimental groups using TRIzol reagent (Invitrogen) and a higher 260/280 ratio was quantified in the NanoDrop 2000 (Thermo Fisher Scientific) as previously reported (39). Briefly, pure RNA (1 ug) was reverse transcripted to cDNA using OligodT and the High Quality RNA-to-cDNA kit (Applied Biosystems). Quantitative Real-Time PCR (qPCR) was done in duplicates, using the Step-One plus RT-PCR system (Life Technology) and the SYBR Green PCR Master Mix (Applied Biosystems). The primers were the following: 18S: (F) 5’-GTAACCCGTTGAACCCCATT-3’ and (R) 5’-CCATCCAATCGGTAGTAGCG-3’; Fasn: 5’-GCTGCGAAACTTCAGGAAAT-3’ and (R) 5’-AGAGACGTGTCACTCCTGGACTT-3’; Scd1: 5’-CCTTCCCCTTCGACTACTCTG-3’ and (R) 5’-GCCATGCAGTCGATGAAGAA-3’. The analysis was normalized to the housekeeping gene 18S and we quantified the RNA expression using the 2-ΔΔCT method for relative quantification. The quantitative analysis is shown in graphs (A) Fasn and (B) Scd1. The graphs are expressed as mean ± S.E.M. (the fold of change relative to the control), n= 4 - 5 per group. The statistical difference is indicated by * compared to Sal+Water (P< 0.05). Fasn: fatty acid synthase and Scd1: Stearoyl-CoA 9-desaturase. [file Image_2.jpeg]

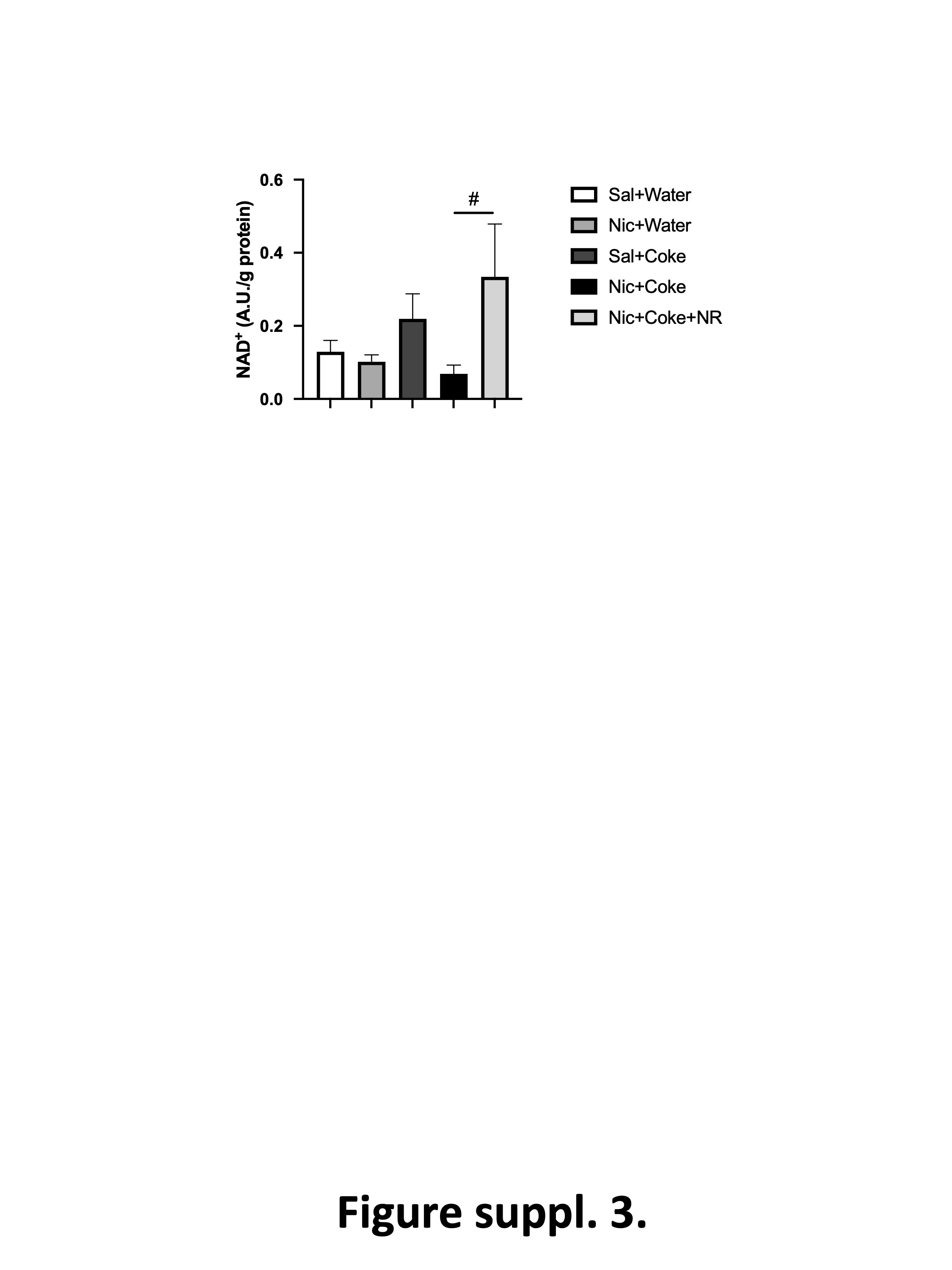

Supplement: Supplementary Figure 3 — NR supplementation increases the NAD+ concentration in the liver. NAD+ levels were analyzed using the commercial EnzyChrom™ NAD/NADH Assay kit (BioAssay Systems, CA, USA) according to manufacturer protocol. NAD+ levels were normalized by liver weight in grams. The graph is expressed as the mean ± S.E.M. n= 3 - 5 per group. The statistical difference is indicated by # compared to Nic+Coke (P< 0.05). [file Image_3.jpeg]

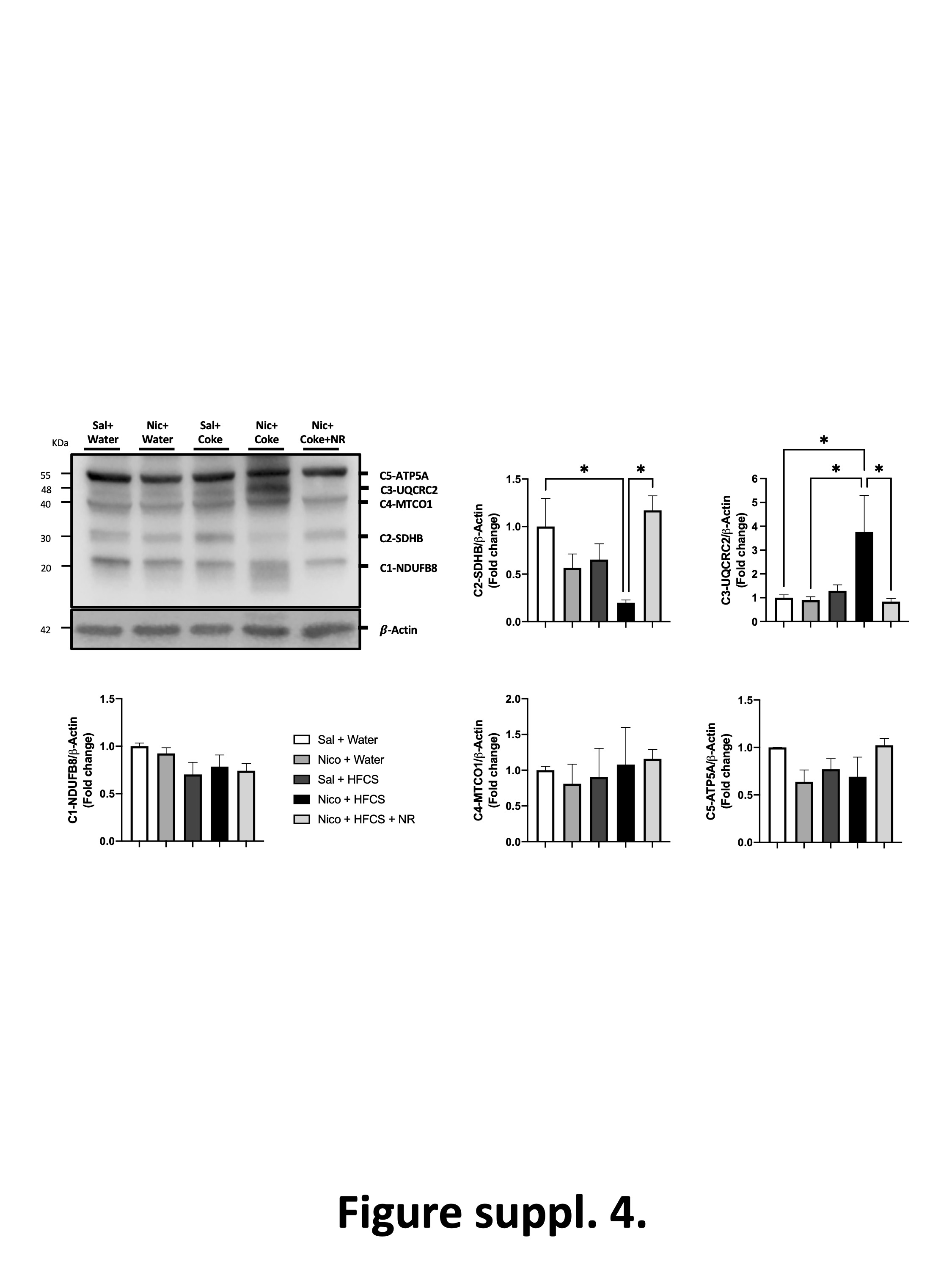

Supplement: Supplementary Figure 4 — Electron transporter protein levels. The liver was excised and homogenized to evaluate electron transporter chain (ETC) proteins from complex 1 to 5 by western blot. Representative western blot image of ETC. β-Actin levels are shown as a loading control. Molecular weight markers are depicted in KDa. The quantitative analysis of the western blot is shown for Complex 1 to 5. The protein levels were normalized to β-Actin and expressed as the mean ± S.E.M. (the fold of change relative to the control), n= 5 - 6 per group. Statistical difference is indicated by * compared to Sal+Water (P< 0.05), & compared to Nic+ Water (P<0.05), and # compared to Nic+Coke (P< 0.05). [file Image_4.jpeg]
