# Supplementary material for: Hepatic steatosis induced by nicotine plus Coca-Cola™ is prevented by nicotinamide riboside (NR)
Source: Front Endocrinol (Lausanne). 2024 May 2;15:1282231. doi: 10.3389/fendo.2024.1282231 (PMC11097688; doi:10.3389/fendo.2024.1282231)
Supplement: Supplementary Table 1 — Treatment injection schedule. Each injection was 200 μl. [file Table_1.docx]

Supplemental Table 1. Treatment injection schedule. Each injection was 200 μl.

|  | Morning injection | | Evening injection | |
| --- | --- | --- | --- | --- |
| Groups | **Nic (0.75 mg/kg)** | **NR (200 mg/kg BW/day)** | **Nic (0.75 mg/kg)** | **NR (200 mg/kg BW/day)** |
| Sal+Water | Sal | Sal | Sal | Sal |
| Nic+Water | Nic | Sal | Nic | Sal |
| Sal+Coke | Sal | Sal | Sal | Sal |
| Nic+Coke | Nic | Sal | Nic | Sal |
| Nic+Coke+NR | Nic | NR | Nic | NR |
