# Supplementary material for: Hepatic steatosis induced by nicotine plus Coca-Cola™ is prevented by nicotinamide riboside (NR)
Source: Front Endocrinol (Lausanne). 2024 May 2;15:1282231. doi: 10.3389/fendo.2024.1282231 (PMC11097688; doi:10.3389/fendo.2024.1282231)
Supplement: Supplementary Table 2 — Scoring for rodent NAFLD (35). [file Table_2.docx]

Supplemental Table 2. Scoring for rodent NAFLD (35).

| **Histological feature** | **Score** | | | |
| --- | --- | --- | --- | --- |
| **Steatosis** | **0** | **1** | **2** | **3** |
| Macrovesicular steatosis | <5% | 5-33% | 33-66% | >66% |
| Microvesicular steatosis | <5% | 5-33% | 33-66% | >66% |
| Hypertrophy | <5% | 5-33% | 33-66% | >66% |
| **Inflammation** |  |  |  |  |
| Number of inflammatory foci/field | <0.5 | 0.5-1.0 | 1.0-2.0 | >2.0 |
